# DNA methylation and demethylation dynamics

**DOI:** 10.18632/oncotarget.6039

**Published:** 2015-10-08

**Authors:** Francesco Neri, Danny Incarnato, Salvatore Oliviero

**Affiliations:** HuGeF and Department of Life Sciences and System Biology University of Torino, Italy

**Keywords:** DNA methylation, promoter demethylation, MAB-seq, 5fC, 5caC

DNA methylation is one of the most studied epigenetic modifications and is essential for mammalian development. The methylation pattern is established through an extremely orchestrated mechanism that implicates *de novo* methylation, maintenance of the methylated cytosine, and demethylation. DNA methylation is catalyzed by the well-characterized DNA methyltransferase family enzymes (DNMTs), including the DNMT3A and DNMT3B that are responsible for the *de novo* methylation and DNMT1 that is the mediator of the maintenance of the DNA methylation pattern through cell replication [1]. In contrast, the mechanisms by which removal of DNA methylation occurs remained elusive, until recently when it has been proposed an active mechanism of DNA demethylation involving ten-eleven translocation methylcytosine dioxygenase (TET) and thymine DNA glycosylase (TDG) proteins [2]. The rules that govern the dynamics of DNA methylation/demethylation have not yet been clarified and represent a key need to deeply understand both normal development and diseases. The first step toward a deeper comprehension of the role of 5mC and its oxidized derivatives is the mapping of such modifications on a genome-wide scale. In a recent report, we presented a novel high-throughput method, named Methylation Assisted Bisulfite sequencing (MAB-seq) [3], that enables genome-wide mapping at single-base resolution, and quantitation, of 5fC/5caC residues. MAB-seq is based on the protection of unmethylated cytosines through an *in vitro* methylation treatment, followed by bisulfite conversion, which results in the deamination of only 5fC/5caC residues. Major strength of MAB-seq is the ability to directly map with a single treatment, and in a single sequencing run both 5fC/5caC intermediate products of cytosine demethylation, reducing the need for a higher coverage, typical of subtractive methods [2], and the risk of introducing false positives. Mapping the 5fC/5caC distribution across the genome of mouse embryonic stem cells (ESCs) revealed a strong enrichment of these modifications on enhancers, exons, and repetitive regions, in agreement with previous reports based on affinity purification methods [4, 5]. Surprisingly, MAB-seq also revealed a strong enrichment of 5fC/5caC on active promoters, especially on the transcriptional start sites (TSSs) of actively transcribed genes marked by H3K4me3. This enrichment, and the consequent depletion of 5mC/5hmC marks, becomes more pronounced with the increased level of promoter accessibility, and reveals the existence of active methylation/demethylation cycles occurring on active promoters. As additional evidence, ChIP-seq analysis of Tdg revealed strong co-occupancy of Tdg and Tet1 on the TSSs of H3K4me3 gene promoters, and knockdown of TDG resulted in a significant increase of 5fC/5caC levels on the TSSs of actively transcribed genes.

**Figure 1:**
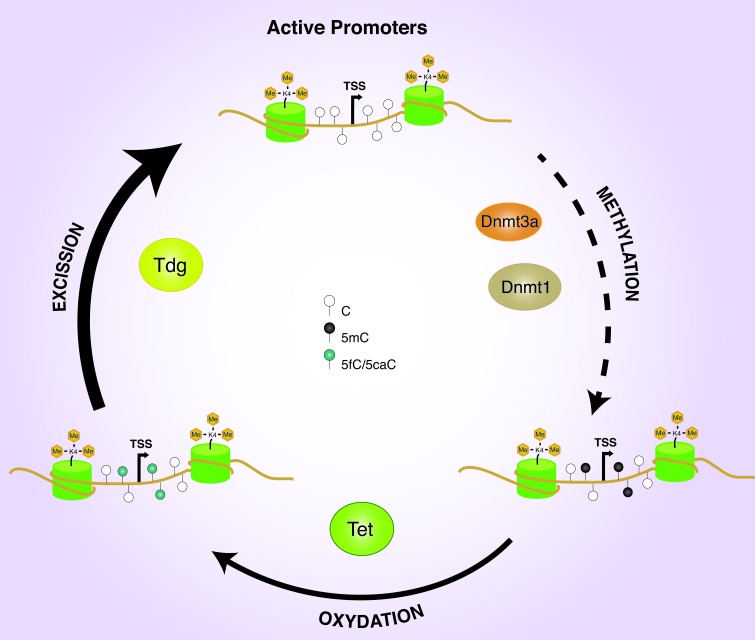
Promoter DNA methylation dynamics H3K4me3 active promoters undergo to DNA methylation and demethylation processes mediated by DNMTs and TET/TDG enzymes.

Moreover, Tet1/2 double knockdown increases the 5mC level on the TSSs of actively transcribed genes, whereas Dnmt1 and Dnmt3a, but not Dnmt3b, knockdown leads to a significant reduction of the methylated cytosine. All together, these data suggest the existence of an articulate circuitry involving Dnmt1/3a, Tet1/2, and Tdg proteins, aimed to maintaining the hypomethylated state of the promoter of the H3K4me3 genes in ESCs (Figure 1).

This evidence shed new light on the mechanism of transcriptional gene repression mediated by the DNA methylation. It has been proposed that promoter of highly expressed genes are free from the action of the DNMTs, probably thanks to the inhibitory function of the H3K4me3 and to the steric hindrance of the transcriptional factors. Our result show for the first time that DNA methylation events can occur on the promoters of the open genes, but they are rapidly eliminated by an active DNA demethylation mechanism. These observations demonstrate that the DNA methylation is an epigenetic feature more flexible and dynamic than previously thought and highlight the importance of TET and TDG proteins in the regulation of active genes with both physiological and pathological implications [6]. Indeed, specific gene promoters’ hypermethylation that characterizes differentiated cells and several diseases could be mediated by loss of the mechanism of active (protective) demethylation rather than targeted de novo DNA methylation. The involvement of Dnmt1 in this (sporadic) DNA methylation of the active gene promoters suggests that, in this context, Dnmt1 could make de novo methylation as previously observed [7].
